# Editorial: Next generation B cell targeting therapies in autoimmune diseases

**DOI:** 10.3389/fimmu.2023.1322546

**Published:** 2023-11-09

**Authors:** Rui Li, Jiahui Zhu, Shenyue Wang, Bo Zhang, Linlu Tian, Ying Fu

**Affiliations:** ^1^ Institute of Immunotherapy, Fujian Medical University, Fuzhou, Fujian, China; ^2^ Department of Neurology and Institute of Neurology of First Affiliated Hospital, Fujian Medical University, Fuzhou, Fujian, China; ^3^ Department of Neurology, Perelman School of Medicine, University of Pennsylvania, Philadelphia, PA, United States; ^4^ Fujian Key Laboratory of Molecular Neurology, Fujian Medical University, Fuzhou, Fujian, China; ^5^ Department of Immunology, Harbin Medical University, Harbin, Heilongjiang, China

**Keywords:** B cell targeted therapy, Bruton tyrosine kinase (BTK), CAR (chimeric antigen receptor) T cells, B cell depleting therapies, NMO (neuromyelitis optica), multiple sclerosis, SLE - systemic lupus erythematosus, rituximab

B cells are involved in autoimmune diseases through both antibody-dependent and -independent mechanisms. Broad depletion of B cells using monoclonal antibodies (mAbs) such as anti-CD20 or CD19 were successful for treating some autoimmune immune diseases suggesting that B cells are important disease-mediators and can be a viable therapeutic target. However, some concerns have been raised in the past few years regarding the durability of the treatment and side effects associated with long-term depletion of B cells (1–3). In addition, due to the limited accessibility of monoclonal antibodies to the diseased tissue and the lack of depletion machinery in the affected organ, mAbs may not deplete infiltrated B cells as efficient as the peripheral B cells (4, 5). Last but not least, not all B cells are pathogenic, some may be even protective, so how to target those pathogenic B cells more specifically and sparing those B cells that are disease protective, will be of great interest in this field. Therefore, we organized this Research Topic to solicit studies that can advance our understanding roles of B cells in autoimmune diseases with particular interest in translational and pre-clinical studies that have therapeutic implications.

Under this Research Topic, there are two review articles and two original research articles (**Figure 1**). Furman et al.’s review article summarized recent advances in B-cell targeted therapies in autoimmune disease of the central nervous system including multiple sclerosis (MS), neuromyelitis optica spectrum disorders (NMOSD) and MOG antibodies associated diseases (MOGAD). The authors started with a comprehensive overview of the developmental trajectory of B cells, with their origin in the bone marrow and culminating in their migration to the peripheral. This journey is accompanied with changes in expression patterns of surface antigens relevant to therapeutic interventions. Notably, the multifaceted capabilities of B cells, including their capacity to secrete proinflammatory cytokines and pathogenic autoantibodies, play a pivotal role in instigating neuroinflammatory processes. Equally important are their regulatory functions, which exert a pronounced influence on pathophysiological mechanisms. Furthermore, the article critically assesses clinical trials related to BCDT, including mAbs, and evaluates the emerging category of B-cell modulating agents, particularly Bruton’s tyrosine kinase (BTK) inhibitors that considered as one of the next generation B-cell targeting therapies. These BTK inhibitors hold considerable promise in the treatment of MS. Compared to BCDT, most BTK inhibitors are able to traverse the blood-brain barrier, allowing them to access the CNS, and hence modulating B cells within the CNS. Additionally, their impact on the myeloid cells renders them capable of addressing compartmentalized inflammation, a critical factor in the propagation of neural damage and the progression of disability in MS.

**Figure 1:**
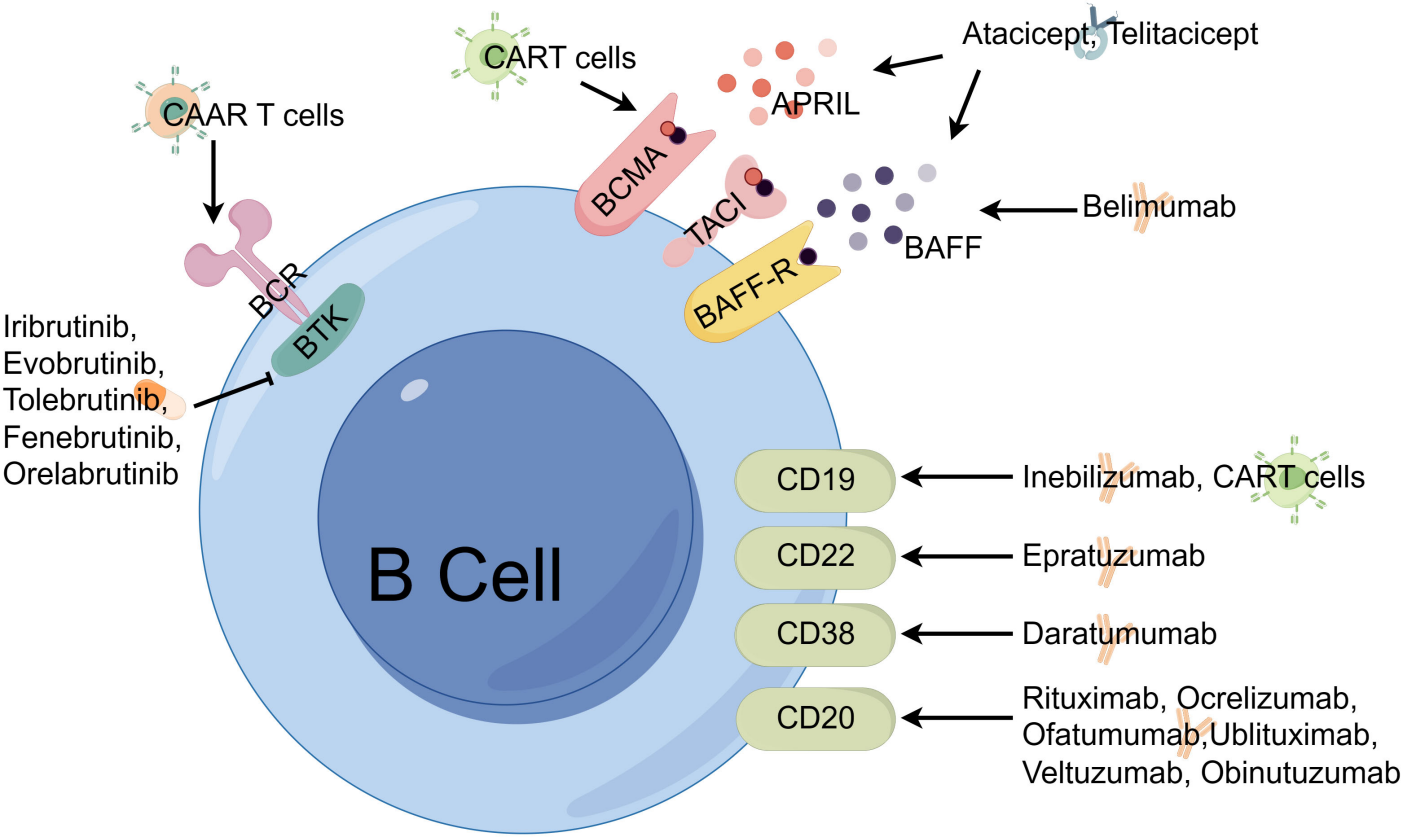
B-cell targeting therapies in autoimmune diseases. APRIL, **(A)** proliferation inducing ligand; BAFF, B cell activating factor; BAFF-R, B cell activating factor receptor; BCMA, B cell maturation antigen receptor; BCR, **(B)** cell receptor; BTK, Bruton’s tyrosine kinase; CAAR, Chimeric auto-antibody receptor; CART cell, Chimeric antigen receptor T cell; CD, Cluster of differentiation; TACI, Transmembrane activator and calcium-modulator and cyclophilin ligand (CAML) interactor. The figure is designed using Figdraw .

In another review article, Zhang et al. provide a thorough overview of BCDT, including mAbs and chimeric antigen receptor (CAR)-based therapies for the treatment of autoimmune diseases. They summarized the mechanisms, effectiveness, safety, and applications of these treatment options while considering the associated merits and drawbacks for patients. These approahes effectively eliminate B cells and/or plasma cells through signaling disruption, complement-dependent cytotoxicity, and antibody-dependent cellular cytotoxicity. Initially applied in the management of hematological malignancies, CAR-based therapy, involves the utilization of engineered T cells to identify and eradicate B cells. Despite their applications in the management of autoimmune diseases, mAbs and CAR-based therapy exhibit several contrasting features. Unlike CAR-based therapy, which requires genetic modification of T cells prior to personal administration, mAbs are readily available and can be employed directly. Moreover, mAbs have been widely used in clinical practice. In contrast, CAR-based therapy for autoimmune diseases is a relatively new therapeutic approach that is currently undergoing research and development. The delicate balance between ensuring the safety and optimizing the efficacy of CAR-based therapy remains a significant concern. Inadequate control of this balance may result in severe and potentially fatal toxicities, notably cytokine release syndrome and neurotoxicity, posing a substantial risk to patients.

One of the research articles conducted by Zhao et al. tried to provide evidence supporting the lower-dose Rituximab (RTX) strategy in treating NMOSD. RTX is commonly recommended as the primary therapeutic option for preventing relapses in neuromyelitis optica spectrum disorder (NMOSD) (6–8). However, establishing a consensus on the optimal dosage regimen remains challenging, basically due to its off-label usage and the lack of large-scale prospective randomized controlled trials (9, 10). Zhao et al. introduces an innovative, low-dose RTX (LD-RTX) strategy, wherein NMOSD patients receive an initial induction therapy with 100 mg of RTX administered once weekly for three weeks, followed by maintenance therapy involving 100 mg RTX reinfusions every six months. This LD-RTX regimen has shown notable efficacy in reducing clinical relapses and mitigating disability among NMOSD patients. Importantly, the study did not record any serious side effects, and none of the subjects had to discontinue treatment due to RTX-related side effects. By endorsing the LD-RTX regimen for NMOSD, which is not only effective but also more affordable and potentially decreasing the risk of unfavorable immunosuppressive effects, this study reopens the discussion on the optimal dosage and timing of treatment for NMOSD patients.

Finally, the research by Weißenberg et al. describes the diminished responsiveness of B cells in systemic lupus erythematosus (SLE) as well as memory B cells in RA and primary Sjögren’s syndrome (pSS) concerning B cell receptor (BCR) and Toll-like receptor 9 (TLR9) signaling. This diminished responsiveness appears to be a status of post-activation functional anergy, potentially induced by *in vivo* BCR engagement without concurrent co-stimulation from T cells. The study emphasizes the pivotal role of CD40 activation in overcoming this reduced responsiveness and restoring proper BCR response. Their research suggests that inhibiting CD40/CD154 interactions could be a potential therapeutic approach to induce B-cell anergy and subsequently reduce disease activity. In support of this hypothesis, another investigation has demonstrated the possibility of using an anti-CD154 antibody to prevent the generation of antibody-secreting cells in SLE patients.

In summary, the Research Topic includes a collection of enlightening articles that consolidate recent breakthroughs in B-cell-directed therapies for autoimmune diseases, while also offering a forward-looking perspective on the potential directions of such therapies in the future. The success of B-cell targeting therapies in managing autoimmune diseases over the past years has underscored the significance of B cells in these conditions and the promise of B-cell-directed treatment strategies. The therapeutic landscape is constantly evolving, driven by our deepening understanding of underlying mechanisms, the refinement of existing therapeutic approaches, and the discovery of novel therapeutic targets.

## Author contributions

RL: Writing – original draft, Writing – review & editing. JZ: Writing – original draft. SW: Writing – original draft. BZ: Writing – review & editing. LT: Writing – review & editing. YF: Writing – review & editing.
